# Surgical Management of Isolated Zygomaticomaxillary Complex Fractures: Role of Objective Morphometric Analysis in Decision-Making

**DOI:** 10.3390/cmtr18040050

**Published:** 2025-11-29

**Authors:** Saša Mijatov, Ivana Mijatov, Denis Brajković, Dušan Rodić, Jagoš Golubović

**Affiliations:** 1 Faculty of Medicine, University of Novi Sad, Hajduk Veljkova 3, 21000 Novi Sad, Serbia; 2 Department of Maxillofacial and Oral Surgery, University Clinical Centre of Vojvodina, Hajduk Veljkova 1, 21000 Novi Sad, Serbia; 3 Department of Neurosurgery, University Clinical Centre of Vojvodina, Hajduk Veljkova 1, 21000 Novi Sad, Serbia

**Keywords:** zygomaticomaxillary complex (ZMC), facial trauma, morphometric analysis, 3D imaging, surgical navigation, facial symmetry, photogrammetry, AI

## Abstract

Zygomaticomaxillary complex (ZMC) fractures are among the most common midfacial injuries, with significant implications for both function and facial esthetics. Optimal management requires restoring the normal anatomical alignment and symmetry of the zygomatic region to prevent long-term deformity and functional deficits. However, the decision-making surrounding surgical intervention, particularly in isolated ZMC fractures with moderate displacement, remains nuanced. This review discusses contemporary surgical approaches for isolated ZMC fractures and examines how objective morphometric analysis can guide critical decisions such as the timing of surgery, choice of surgical approach, and extent of fixation. Conventional assessment tools like computed tomography (CT), cephalometric measurements, and intraoperative imaging provide foundational data on fracture anatomy. Emerging technologies, including three-dimensional (3D) photogrammetry, stereophotogrammetry, artificial intelligence (AI)-based symmetry analysis, and surgical navigation systems, offer advanced means to quantify facial symmetry and bone alignment. By integrating these objective metrics into clinical practice, surgeons can enhance preoperative planning and postoperative outcome evaluation, with a particular focus on achieving facial symmetry for optimal esthetic and functional results. We also outline clinical decision-making frameworks that incorporate quantitative measurements, and we discuss current limitations, future directions, and the potential for standardizing protocols in the management of ZMC fractures.

## 1. Introduction

The zygomaticomaxillary complex (ZMC) forms the prominence of the cheek and a key structural buttress of the midface. Isolated fractures of the ZMC (often referred to as “tripod” fractures) are common facial injuries commonly caused by blunt trauma (e.g., motor vehicle accidents, assaults, or falls) [1,2]. These fractures involve disruption of the zygomatic bone’s articulations with the axilla, frontal bone, temporal bone (zygomatic arch), and often the orbital floor or lateral orbital wall. Because the zygoma largely defines cheek width and contour, ZMC fractures readily lead to noticeable facial asymmetry, cheek flattening, and associated functional problems [3]. Patients may present with malar flattening (loss of cheek prominence), periorbital swelling and bruising, infraorbital nerve numbness in the cheek and upper lip, trismus (limited jaw opening when a displaced zygomatic arch impinges on the coronoid process), diplopia (double vision) from orbital floor disruption causing globe malposition, or cosmetic concern over facial deformity. The primary goal of treatment is to restore normal anatomy-re-establishing the proper alignment of the zygoma in all planes thereby correcting deformity and preventing long-term functional sequelae (such as persistent visual or jaw dysfunction). In cases of grossly displaced fractures, the indication for surgical repair is clear, as these injuries will not heal in an acceptable position on their own [3,4]. However, many ZMC fractures are only moderately displaced, making the decision to operate less straightforward. Traditionally, clinical examination findings (such as palpable bony step-offs, degree of malar depression, and ocular motility or gaze symmetry) combined with surgeon experience have guided management. Facial swelling in the acute phase can mask subtle asymmetries or misalignment, so fractures that appear minor initially may still warrant surgery after swelling subsides [5,6]. This uncertainty has driven interest in using objective morphometric criteria to better guide such decisions. By quantitatively measuring how far the zygoma has shifted from its normal position or how asymmetrical the facial skeleton has become, clinicians can make more evidence-based judgments regarding the need for surgery and its urgency [3,4,7]. Concurrently, advances in imaging and computer-assisted technologies provide new opportunities to plan and execute repairs. This review will explore the spectrum of surgical approaches for isolated ZMC fractures and delve into how objective measurements: from CT scans, 3D surface imaging, and other modalities can influence the timing of intervention, the surgical technique chosen, and the assessment of outcomes [4,8,9]. We will also discuss the importance of facial symmetry in evaluating success, and how emerging tools and standardized protocols may shape the future of ZMC fracture management.

## 2. Materials and Methods

A comprehensive literature review was conducted to gather current information on the management of isolated ZMC fractures and the use of morphometric analysis in clinical decision-making. Sources included peer-reviewed journals in maxillofacial and plastic surgery, as well as relevant craniofacial surgery textbooks and practice guidelines. We searched databases (PubMed, Scopus) for articles published in the last two decades, with emphasis on studies from approximately 2010 onward that introduced new imaging techniques or quantitative assessment tools for facial fractures. Because this is a narrative review rather than a systematic meta-analysis, we did not compile or report detailed counts of identified, screened, and included articles; instead, we sought to capture a broad overview of the pertinent literature. Key search terms included combinations of “zygoma” or “zygomaticomaxillary complex fracture” with words like “surgical management,” “CT analysis,” “facial symmetry,” “3D photogrammetry,” “stereophotogrammetry,” “artificial intelligence,” and “surgical navigation.” Additional references were obtained by screening the bibliographies of key articles. Both clinical studies (retrospective analyses, prospective trials, case series) and technical papers on novel technologies were included to ensure a broad perspective. The gathered information was synthesized into a narrative review, structured by thematic topics as presented below.

## 3. Results and Discussion

### 3.1. Surgical Approaches for Isolated ZMC Fractures

The surgical strategy for ZMC fractures is to achieve anatomical reduction in the displaced bone and secure it with stable fixation. In general, significantly displaced or unstable fractures require open reduction and internal fixation (ORIF), ideally performed within 7–14 days post-injury (after acute swelling subsides but before bone healing begins). Conversely, minimally displaced fractures without functional deficits may be managed conservatively with observation, though close follow-up is essential to ensure no late-developing asymmetry or ocular problem [10]. When operative intervention is indicated, a variety of approaches are available to access the zygoma and its articulations. Surgeons choose one or a combination of incisions to adequately visualize fracture sites while minimizing morbidity. Common approaches include:

1. Intraoral (Keen’s) approach: Incision inside the upper lip (buccal sulcus) to reach the zygomaticomaxillary buttress and lateral maxilla, providing a route to elevate and align the malar prominence from below with no external scar [11].
2. Transconjunctival or subciliary approach: Incisions via the lower eyelid (inside the conjunctiva or just below the eyelashes) to access the infraorbital rim and orbital floor, often used when the orbital margin or floor requires repair; these approaches leave either no scar or a well-concealed scar [11,12].
3. Lateral brow or upper eyelid approach: A small incision in the lateral eyebrow region or within the upper eyelid crease to expose the frontozygomatic suture (lateral orbital rim) for fracture reduction and plating; this approach avoids a visible scar on the forehead [13].
4. Temporal (Gillies) approach: A short incision behind the hairline in the temple, allowing insertion of an instrument to lever and elevate a depressed zygomatic arch. This minimally invasive technique is effective for isolated arch displacement and is often adjunctive to other exposures [14].
5. Coronal (hemicoronal) approach: A longer incision across the scalp behind the hairline for wide access to the zygoma, orbital rims, and arch. Reserved for comminuted or complex fractures, it provides excellent visualization at the cost of a more extensive dissection [15].

Using two or more of these approaches in combination is common for comprehensive access (for example, combining an intraoral and a transconjunctival incision to cover both the inferior and superior aspects of the ZMC). Once the fragments are exposed and mobilized, the surgeon reduces the zygoma to its correct position, often using the intact opposite side as a template for symmetry. Rigid fixation is then applied with small plates and screws at strategic locations. A stable tripod fixation usually involves at least two or three points-typically the zygomaticofrontal suture, infraorbital rim, and zygomaticomaxillary buttress-to ensure the bone maintains its proper alignment. In less severe fractures, sometimes one well-placed plate can suffice if the other articulations realign spontaneously. If the orbital floor is fractured and the globe is at risk of herniation or sagging, an orbital floor exploration and graft or implant repair is performed during the same surgery. The net goal is to restore the facial contour and projection of the cheekbone to its pre-injury state [16,17,18].

### 3.2. Conventional Imaging and Morphometric Assessment

Accurate assessment of a ZMC fracture begins with high-quality imaging. Computed tomography (CT) is the gold standard for evaluating midfacial fractures, offering detailed visualization of fracture lines, fragment displacement, and any impingement on adjacent structures. Thin-slice axial CT with coronal and sagittal reconstructions, as well as 3D reconstructions, enables the surgeon to understand the extent of displacement and rotation of the zygomatic bone [3]. (Traditional skull radiographs, such as Waters’ or submentovertex views, were historically used but are now largely obsolete due to the superior detail provided by CT).

CT also allows for morphometric analysis: the quantification of fracture displacement using measurable distances and angles. In practice, surgeons often compare key anatomic reference points on the injured side versus the uninjured side. For example, the lateral position of the zygoma can be gauged by measuring the distance from the zygomatic lateral wall to a fixed midline landmark on each side; a significant discrepancy indicates medial displacement of the fractured zygoma. Similarly, vertical depression can be assessed by noting any difference in orbital floor height or zygomatic arch position between sides. These objective measurements give a sense of how far off the bone is from its normal alignment. Even simple linear metrics, taken on standard CT images or 3D models, can stratify fracture severity: a few millimeters of displacement might fall within acceptable bounds for conservative management, whereas larger differences (e.g., more than 4–5 mm inward collapse or >2–3 mm posterior shift) typically portend noticeable asymmetry or functional issues and thus favor operative reduction. Intraoperatively, some surgeons will also obtain a quick CT or use a fluoroscopic C-arm to verify that the major components of the ZMC are properly reduced before finalizing fixation. This immediate imaging feedback can reveal subtle malpositions, for instance, a slight residual rotation or a small step at a suture line allowing for correction on the spot and thereby reducing the likelihood of postoperative surprises or the need for revision [19,20,21].

### 3.3. Modern 3D Technologies and Quantitative Tools

Emerging technologies are expanding the surgeon’s toolkit for analysis and precision. 3D photogrammetry (including stereophotogrammetry) enables capture of the patient’s facial surface in three dimensions using specialized cameras or even smartphone sensors. The resulting 3D facial model can be used to quantify asymmetry pre- and postoperatively. By superimposing the mirror image of the uninjured side onto the injured side, one can generate color-coded maps of surface discrepancies and calculate metrics such as mean absolute differences. This provides an objective assessment of facial contour restoration often revealing subtle depressions or protrusions that may not be obvious by eye. Because it is noninvasive and uses no radiation, serial photogrammetric analysis is feasible for tracking healing and the effectiveness of interventions [22,23].

In addition, Artificial Intelligence (AI) techniques are being explored in facial fracture management. AI algorithms can automatically detect fractures on imaging and perform symmetry analysis by identifying key facial landmarks. They can generate a quantitative symmetry score or highlight subtle asymmetries that might escape the human eye. Looking ahead, AI-based planning may virtually mirror the patient’s intact side onto the fractured side to suggest optimal bone repositioning, providing a data-driven surgical blueprint for the operation [24,25,26].

Intraoperative computer-assisted navigation has also become an important adjunct in complex ZMC fracture repairs. Navigation systems use the patient’s preoperative CT data and track surgical instruments or the patient’s anatomy in real time to guide the surgeon. By registering the operative field to the CT scan, the surgeon can probe a point on the zygoma and see its location relative to the desired position on a monitor. This is particularly valuable when normal landmarks are unreliable (such as in comminuted fractures) or when high precision is essential (for example, aligning the orbital floor and lateral wall to prevent any discrepancy in globe position). Navigation can verify that a reduction achieves symmetry, for example, confirming the restored malar prominence matches the contralateral side within a small margin of error. The trade-off is that navigation requires extra setup, specialized equipment, and a learning curve to use effectively. It is often employed in tertiary centers for the most challenging cases, and its usage is likely to grow as the technology becomes more streamlined [27,28].

### 3.4. Integration of Objective Metrics in Planning and Outcome Evaluation

**Figure 1:**
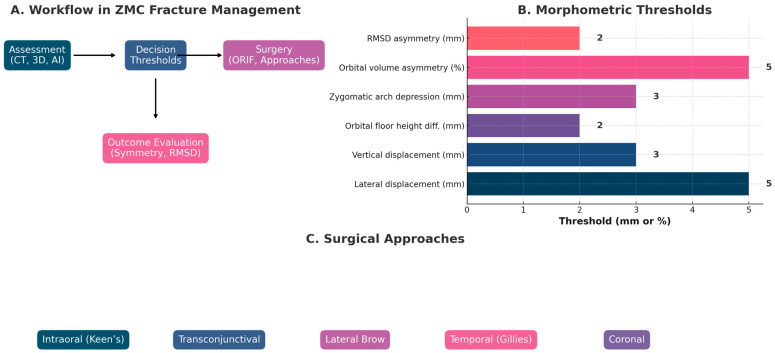
ZMC composite chart with objective metrics and fracture management protocols.

Influence on surgical planning: The integration of objective measurements begins at the stage of deciding whether and when to operate. Quantified displacement on imaging can tilt the balance in equivocal cases. For example, if a CT scan analysis shows that the zygoma is displaced only 2 mm from its normal position and the orbit remains properly aligned, a surgeon may opt for close observation rather than immediate surgery, confident that any asymmetry is within a tolerable range. On the other hand, if measurements reveal a 5–6 mm medial collapse of the zygomaticomaxillary buttress and significant rotation of the zygoma, this serves as an objective trigger for intervention even if outward deformity seems moderate. Objective morphometric metrics with clinical relevance are given in Table 1 and Figure 1. These metrics can prevent under-treatment of fractures that would likely result in cosmetic or functional problems if left alone. They also inform the surgical approach: knowing the extent and direction of displacement helps in choosing which incisions and maneuvers will best access and correct the deformity. For instance, a measured inferior sag of the zygoma might prompt an approach that allows lifting the bone (like combining an intraoral with an orbital floor approach), whereas an isolated arch inward bowing measured on CT might be resolved with a targeted temporal approach alone. Objective preoperative analysis can even guide the expected amount of force or translation needed to reposition the bone, and highlight if adjunct procedures (such as orbital floor reconstruction) are necessary by assessing changes in orbital volume or rim alignment [29,30,31]. Where numerical thresholds are provided (e.g., >4–5 mm lateral displacement or >2 mm orbital floor height differences), these values originate from a combination of large retrospective cohorts, clinical scoring systems, and smaller prospective studies. Although widely cited, their evidence levels vary, and several thresholds are supported primarily by observational data rather than randomized evidence. Similarly, AI-based symmetry tools remain dependent on dataset quality, training variability, and may exhibit reduced interpretability (“black-box” effect). Surgical navigation, while offering sub-millimetric spatial guidance, is limited by registration error accumulation and center-specific expertise. These considerations should be recognized when applying threshold-based decision support in clinical practice.

Influence on outcome evaluation: After treatment, objective metrics provide a means to rigorously evaluate the success of fracture reduction. Rather than relying solely on subjective visual assessment, surgeons can use post-reduction CT or 3D scans to compare the rebuilt anatomy with the original or with the contralateral normal side. Techniques like mirroring the healthy side onto the injured side allow calculation of any residual discrepancies. Quantitative indices such as the root mean square distance (RMSD) between the two sides of the face can summarize overall symmetry. Postoperative analysis might show, for example, that the cheek prominence on the injured side is within 2 mm of the uninjured side. Such quantifiable symmetry strongly correlates with a normal appearance from the patient’s perspective. Facial symmetry is not just a cosmetic ideal; it has functional correlates as well. Symmetric zygomatic positions help ensure balanced occlusion and temporomandibular joint function, and symmetrical orbits help maintain proper binocular visual alignment. By documenting outcomes with objective data, surgeons can identify any residual areas that might require refinement (such as a plate adjustment or additional contouring in a secondary procedure). Furthermore, pooling such data across patients enables analysis of which surgical techniques yield the best symmetry, thus driving evidence-based improvements in care. In summary, objective morphometric evaluation closes the feedback loop by verifying that the goals of surgery-restoring form and function have been met to a high standard of precision. However, surgeons must recognize that clinical facial symmetry is ultimately expressed at the soft-tissue level, not at the skeletal level alone. Soft-tissue adaptation after trauma or surgery is influenced by patient-specific variables such as age, skin elasticity, muscular tone, subcutaneous fat distribution, and healing capacity. These factors may produce delayed contour changes even after an anatomically accurate bony reduction. Therefore, objective skeletal morphometry should be interpreted in conjunction with expected soft tissue behavior to better predict final aesthetic and functional outcomes [25,32,33].

### 3.5. Towards an Objective Decision-Making Framework

The trend toward evidence-based thresholds is inspiring the development of formal decision-making frameworks for ZMC fractures. Rather than relying solely on subjective judgment or variable heuristics, surgeons are beginning to incorporate specific metric criteria into their algorithms for care (Table 2). Recent studies have identified quantitative cutoff values (for example, a defined number of millimeters of medial wall impaction or arch depression) that strongly correlate with the need for surgery. Incorporating such cutoffs into practice means that if a fracture’s measurements exceed those limits, operative intervention is recommended, whereas if all measurements are below thresholds and the patient is asymptomatic, a nonoperative approach with observation can be justified. This method helps reduce ambiguity in moderate cases and ensures that patients with significant deformity are not missed. Essentially, objective measurements provide a safety net and a common language: two surgeons evaluating the same CT data are more likely to agree on management if clear numeric guidelines are in place [34].

These quantitative criteria are meant to complement, not replace, traditional clinical assessment and classification systems. Established classifications (like the Zingg or AO systems) categorize fractures by pattern and complexity. By overlaying objective data onto these categories, one can refine the treatment plan. For instance, a ZMC fracture classified as “moderate” by pattern might still be managed aggressively if the measured orbital floor drop or zygomatic displacement is high. Conversely, a fracture appearing severe on imaging might be managed more conservatively if measurements reveal only minimal actual shift and the patient has no functional deficit. Some experts envision a point-based scoring system that tallies various factors like displacement in different planes, degree of comminution, soft tissue involvement, and clinical symptoms to yield a score guiding treatment recommendations. Such tools could be especially useful in training environments and in busy trauma centers to standardize decision-making. As outcome data accumulate, professional guidelines are likely to evolve accordingly. In the future, we may see consensus statements advising that, for example, “a ZMC fracture with >5 mm displacement in any plane or significant asymmetry on 3D analysis should undergo operative repair,” thus providing a clear, evidence-backed rule. The overarching goal is to marry quantitative rigor with clinical acumen, improving consistency and outcomes in ZMC fracture management across the board [8,14,35,36].

### 3.6. Current Limitations and Future Directions

Despite the promise of objective morphometric analysis, several limitations currently prevent its universal adoption. High-end imaging equipment (such as intraoperative CT scanners and optical 3D surface cameras) and surgical navigation systems are not available in all hospitals, especially outside major centers, due to cost and infrastructure requirements. Even when such tools are available, they can introduce additional complexity and require a learning curve for the surgical team. Another challenge lies in standardization: different surgeons or institutions might measure displacement using varying reference points, potentially leading to inconsistent assessments. This underscores the need for establishing uniform measurement protocols if objective analysis is to be widely implemented. Additionally, while we can measure asymmetry with sub-millimeter precision, not all differences are clinically significant. More research is needed to determine the thresholds of asymmetry that correlate with patient-perceived outcomes—essentially, how much difference is too much. Similarly, AI-driven software and advanced analysis tools, although promising, are still being refined and must be validated across diverse patient populations and imaging conditions to ensure their recommendations are reliable [37,38,39].

Looking ahead, continued technological and clinical advancements are likely to mitigate these limitations. Just as computing power and imaging capabilities have surged over the past decade, we can anticipate more accessible solutions for morphometric analysis. For instance, compact handheld 3D scanners or even smartphone-based depth cameras may soon enable quick facial scans in the clinic, bringing photogrammetry to virtually any practice. Augmented reality (AR) tools are being explored that could project guidance overlays onto the patient during surgery, effectively merging the preoperative plan (and its objective measurements) with the surgeon’s view in real time. Custom 3D-printed surgical guides or patient-specific implants—designed using precise CT measurements and mirrored anatomy represent another frontier for ensuring that reductions are executed exactly as planned. Furthermore, ongoing research will better define the relationship between measured asymmetries and quality of life, helping to fine-tune what the target metrics should be. As evidence accumulates, professional bodies in cranio-maxillofacial surgery are likely to develop consensus recommendations that incorporate objective metrics into treatment guidelines. In summary, while current use of these technologies is somewhat limited to specialized centers, the trajectory points toward broader adoption. With improvements in cost, user-friendliness, and clinical validation, objective morphometric analysis is poised to become a standardized component of managing ZMC fractures in the future [14,40,41].

## 4. Conclusions

The management of isolated zygomaticomaxillary complex fractures is evolving from an experience-driven art toward a data-informed science. Surgical skill and judgment remain paramount, but objective morphometric analysis now plays a critical adjunct role in guiding that expertise toward optimal outcomes. By measuring fracture displacements on imaging, employing advanced tools like 3D photogrammetry and AI-driven analysis, and utilizing intraoperative navigation or imaging, surgeons can achieve more accurate reductions and thereby restore facial symmetry with greater confidence. These technologies supplement the surgeon’s clinical assessment by providing quantitative benchmarks for when to operate, how to execute the repair, and how to evaluate the result. Patients ultimately benefit from this precision through improved aesthetic and functional outcomes and potentially fewer secondary corrections due to undetected misalignments. The trajectory of craniofacial trauma care is clearly toward greater integration of objective metrics. Future standard protocols will combine the understanding of experienced surgeons with the precision of quantitative data. In summary, objective morphometric analysis is a valuable adjunct to surgical management, helping surgeons reliably restore facial form and function to pre-injury state.

## Figures and Tables

**Table 1:** Objective morphometric metrics with clinical relevance thresholds.

Metric	Clinical Relevance
Lateral displacement (mm)	>4–5 mm displacement often requires surgical correction
Vertical displacement (mm)	>2–3 mm vertical drop associated with noticeable asymmetry
Orbital floor height difference (mm)	>2 mm difference risks diplopia or enophthalmos
Zygomatic arch depression (mm)	Impairs mandibular motion (trismus); >3 mm usually treated
Orbital volume asymmetry (%)	>5% asymmetry linked to cosmetic and functional issues
Root mean square distance (RMSD, mm)	Quantifies global facial asymmetry; <2 mm difference considered acceptable

**Table 2:** Conventional vs. modern tools in ZMC fracture assessment (advantages and limitations).

Tool/Technique	Advantages	Limitations
CT (Computed Tomography)	Gold standard; detailed visualization of fractures; widely available	Radiation exposure; cost; requires expertise
CBCT (Cone-beam CT)	Lower radiation dose; high spatial resolution for facial skeleton	Limited soft tissue resolution; smaller field of view
Cephalometric Analysis	Simple linear/angular measurements; cost-effective	Two-dimensional; limited accuracy in complex 3D displacements
Intraoperative Imaging	Immediate confirmation of reduction; prevents revision surgery	Additional equipment and cost; increases OR time
3D Photogrammetry	Non-invasive; radiation-free; serial follow-up possible	Requires specialized equipment; cost
Stereophotogrammetry	High accuracy 3D surface capture; reproducible symmetry analysis	Limited availability; requires standardization
AI-based Symmetry Analysis	Automated, objective, detects subtle asymmetry; predictive planning	Still under development; validation needed
Surgical Navigation	Real-time intraoperative guidance; increased precision in reduction	Expensive; steep learning curve; limited to specialized centers

## Data Availability

No new data was created.

## Abbreviations

The following abbreviations are used in this manuscript:

ZMC	Zygomaticomaxillary complex
ORIF	Open reduction and internal fixation
CT	Computed tomography
CBCT	Cone-beam computed tomography
3D	Three-dimensional
AI	Artificial intelligence
MRI	Magnetic resonance imaging
RMSD	Root mean square distance
